# Membranes and Synaptosomes Used to Investigate Synaptic GABAergic Currents in Epileptic Patients

**DOI:** 10.3390/membranes14030064

**Published:** 2024-03-02

**Authors:** Alessandro Gaeta, Lilian Juliana Lissner, Veronica Alfano, Pierangelo Cifelli, Alessandra Morano, Cristina Roseti, Angela Di Iacovo, Eleonora Aronica, Eleonora Palma, Gabriele Ruffolo

**Affiliations:** 1 Department of Physiology and Pharmacology, Sapienza University of Rome, 00185 Rome, Italy; alessandro.gaeta@uniroma1.it (A.G.); lilian.lissner@uniroma1.it (L.J.L.); gabriele.ruffolo@uniroma1.it (G.R.); 2 IRCCS San Raffaele Roma, 00166 Rome, Italy; veronica.alfano@sanraffaele.it; 3 Department of Applied Clinical and Biotechnological Sciences, University of L’Aquila, 67100 L’ Aquila, Italy; pierangelo.cifelli@univaq.it; 4 Department of Human Neuroscience, University of Rome Sapienza, 00185 Rome, Italy; alessandra.morano@uniroma1.it; 5 Department of Biotechnology and Life Sciences, University of Insubria, 21100 Varese, Italy; cristina.roseti@uninsubria.it (C.R.); adiiacovo@uninsubria.it (A.D.I.); 6 Center for Research in Neuroscience, University of Insubria, 21052 Busto Arsizio, Italy; 7 Amsterdam UMC, Department of (Neuro)Pathology, Amsterdam Neuroscience, University of Amsterdam, Meibergdreef 9, 1105 AZ Amsterdam, The Netherlands; e.aronica@amsterdamumc.nl; 8 Stichting Epilepsie Instellingen Nederland, 0397 Heemstede, The Netherlands

**Keywords:** GABA_A_ receptors, electrophysiology, synaptic inhibition

## Abstract

Among the most prevalent neurological disorders, epilepsy affects about 1% of the population worldwide. We previously found, using human epileptic tissues, that GABAergic neurotransmission impairment is a key mechanism that drives the pathological phenomena that ultimately lead to generation and recurrence of seizures. Using both a “microtransplantation technique” and synaptosomes preparations from drug-resistant temporal lobe epilepsies (TLEs), we used the technique of two-electrode voltage clamp to record GABA-evoked currents, focusing selectively on the synaptic “fast inhibition” mediated by low-affinity GABA_A_ receptors. Here, we report that the use-dependent GABA current desensitization (i.e., GABA rundown, which is evoked by applying to the cells consecutive pulses of GABA, at high concentration), which is a distinguishing mark of TLE, is mainly dependent on a dysfunction that affects synaptic GABA_A_ receptors. In addition, using the same approaches, we recorded a depolarized GABA reversal potential in synaptosomes samples from the human epileptic subicula of TLE patients. These results, which confirm previous experiments using total membranes, suggest an altered chloride homeostasis in the synaptic area. Finally, the lack of a Zn^2+^ block of GABA-evoked currents using the synaptosomes supports the enrichment of “synaptic fast inhibitory” GABA_A_ receptors in this preparation. Altogether, our findings suggest a pathophysiological role of low-affinity GABA_A_ receptors at the synapse, especially during the fast and repetitive GABA release underlying recurrent seizures.

## 1. Introduction

Epilepsy is one of the most common brain disorders, affecting millions of people worldwide [1]. Although the majority of epilepsies can be medically treated, a percentage (about 20–30%) of epileptic patients experience drug-resistant seizures [2]. The impairment of γ-aminobutyric acid (GABA_A_) receptors’ function is frequently found in several kinds of epileptic disorders, which is not surprising, since GABA is the most important mediator of inhibitory neurotransmission in the central nervous system (CNS), acting on both ionotropic and metabotropic receptors (GABA_A_Rs and GABA_B_Rs, respectively) [3,4,5,6]. Among these, GABA_A_ receptors (GABA_A_Rs) are heteropentameric ligand-gated channels mostly permeable to Cl^−^ ions [4,7] which are formed by the combination of αβγ/δ subunits [8,9].

Synaptic GABA_A_Rs, which contain mostly α1–3, β1–3, and γ2 subunits, mediate the hyperpolarization of the postsynaptic neuronal membrane [10,11,12], leading to so-called “phasic” inhibition [9,13]. On the other hand, extrasynaptic α4βδ, α5βγ, and αβ isoforms, which are activated by low GABA concentrations, generate “tonic” inhibitory currents [5,14,15,16].

Recently, we showed that the physiological activity of GABA_A_Rs is altered in different types of epilepsy [17,18,19,20], mostly by taking advantage of the technique of microtransplanting membranes into *Xenopus laevis* oocytes. This approach allows us to perform electrophysiological experiments on transplanted human receptors that maintain their native properties [17,19]. However, when using a total-membranes preparation, it was not possible to distinguish between synaptic and extrasynaptic currents except by using specific pharmacological agents able to selectively block some specific subtypes of receptors [21,22,23]. Therefore, in the present study, we used a simple but efficient approach to isolate synaptosomes from temporal lobe epilepsy (TLE) human brains [24,25,26,27]. This method can allow for a more detailed characterization of synaptic GABA_A_Rs’ activity and the possible dysfunction underlying epilepsy.

Previously, we showed that the repetitive stimulation of GABA_A_R can induce a significant GABA-current desensitization (i.e., rundown) in human epileptic brains that was not recorded using control tissues [17,28], which we hypothesized was due to the dephosphorylated state of the GABA_A_Rs [17]. Indeed, this phenomenon of “receptors instability” can be prevented by stimulating tyrosine kinase receptor B (TrKB) with BDNF [29] or inhibiting phosphatase action [17].

Phasic synaptic receptors are also characterized by their low affinity for GABA and low sensitivity to inhibition by Zn^2+^. In contrast, extrasynaptic receptor subtypes possess high affinity, thus showing more sensitivity both to GABA and Zn^2+^ [30,31].

Another open question is whether chloride homeostasis, which is a crucial part of neurodevelopmental processes, is differentially regulated in extrasynaptic or synaptic compartments. Indeed, during their development, the intracellular concentration of chloride is higher in immature neurons, resulting in depolarizing GABA activity, whereas, in mature neurons, the activation of GABA_A_ receptors is followed by an influx of chloride that leads to a hyperpolarizing GABAergic response [32]. It is well-known that intracellular [Cl^−^] homeostasis in neurons is regulated by Na-K-2Cl cotransporter isoform 1 (NKCC1) and the K-Cl cotransporter isoform 2 (KCC2) [7,33,34], with NKCC1 responsible for chloride influx and KCC2 for chloride efflux [12,32]. Impaired [Cl^−^] regulation results in a shift in the GABA reversal potential (E_GABA_) and subsequently determines an imbalance of its excitation/inhibition ratio, as reported in several neurodevelopmental diseases [10,35] such as Dravet Syndrome [19]. Notably, a similar altered [Cl^−^] regulation has been described in the subicula of drug-resistant TLE [36] patients and in neocortical epileptogenic human brain tumours [37]. Here, we investigated the aforementioned pathophysiological mechanisms by comparing the current responses obtained with the microtransplantation of whole membranes to those obtained from the synaptosomes of the same epileptic patients.

## 2. Materials and Methods

### 2.1. Patients

Human brain samples were provided by the Departments of Neuropathology of Amsterdam UMC (Amsterdam, the Netherlands) and the University Medical Center Utrecht (UMCU, Utrecht, the Netherlands). Temporal lobe cortical and hippocampal samples were obtained from drug-resistant epileptic patients who were treated with surgical resection of the epileptic focus (Table 1, the symbol “#” is used to refer to the patients throughout the text). For comparison, in one set of experiments we used a cortical sample (TL, male, aged 31 years; respiratory failure) from one control individual without any neurological disorders. After this procedure, the tissue was snap-frozen in liquid nitrogen without delay and then used to perform the electrophysiology experiments. Tissue was obtained and used in accordance with the Declaration of Helsinki and the Amsterdam UMC Research Code provided by its Medical Ethics Committee and approved by the science committee of the Biobank (21–174).

### 2.2. Membranes and Synaptosomes’ Preparation and Intracellular Injection

Right after their shipment, brain samples were immediately used or stored at −80 °C for later utilization. For total membrane extraction, we performed a standard protocol [17]: the samples were homogenised in membrane buffer solution (200 mM glycine, 150 mM NaCl, 50 mM EGTA, 50 mM EDTA, and 300 mM sucrose, plus 20 µL of protease inhibitors, P2714 by Sigma, pH 9, adjusted using NaOH). Subsequently, we centrifuged the material for 15 min at 9500× *g*. Afterwards, we centrifuged the supernatant for 2 h at 100,000× *g* using a Beckman-Coulter ultracentrifuge. Before aliquoting and storage at −80 °C, the pellet was rinsed with sterile water and re-suspended in 5 mM glycine. Membrane injection into *Xenopus laevis* oocytes and electrophysiological recordings were executed as previously described [17]. With regard to synaptosomes’ isolation, the samples were homogenised in Syn-Per Extraction Reagent [38]. Subsequently, in the first step of centrifugation, the material was centrifuged for 20 min at 1200× *g*. Then, with a Beckman-Coulter ultracentrifuge, the supernatant was centrifuged for 25 min at 15000× *g* to obtain a pellet enriched in synaptosomes.

This pellet was re-suspended in Syn-Per solution, aliquoted, and kept at −80° for later usage. All animal protocols (female *Xenopus laevis* frogs) were approved by the Italian Ministry of Health (no. authorization no 427/2020-PR).

### 2.3. Electrophysiological Recordings

The electrophysiological experiments were carried out using the “two-electrodes voltage-clamp” technique [17], 18–48 h after cytoplasmic injection. The temperature in the laboratory was maintained at 21–23 °C during recordings. The recording chamber (0.1 mL volume) where the oocytes were placed was constantly perfused with oocyte Ringer solution (OR: NaCl 82.5 mM; KCl 2.5 mM; CaCl_2_ 2.5 mM; MgCl_2_ 1 mM; Hepes 5 mM, adjusted to pH 7.4 using NaOH). Here, the cells were clamped with two KCl 3M-filled microelectrodes (or 3M K^+^-acetate). The application of neurotransmitters was controlled with a computer interface, connected to a gravity-driven multi-valve perfusion system (9–10 mL/min) (Biologique RSC-200, operated by its dedicated software; Claix, France). These valves were open or closed using digitally set intervals, thus determining the interruption or initiation of the solution’s flow in the polyethylene tubes connected to the recording chamber (Harvard Apparatus, Cambridge, MA, USA). Before the experiments, we evaluated the stability of GABA-evoked currents by applying two pulses of GABA (500 µM), separated by a 4 min washout. After this test, only the cells that displayed a <5% variation of current amplitude were used for further recordings (i.e., rundown, E_GABA_, and inhibition by zinc, as detailed below). The GABA current reversal potential (E_GABA_) was calculated using a current–voltage (I–V) curve and then elaborated by a linear regression curve-fitting software (Sigmaplot 15). GABA current rundown was measured by the application of six pulses of GABA 500 μM for 10 s, each separated by a 40 s wash. The GABA current’s decrease (i.e., rundown) was expressed as the percentage of residual GABA current after the whole protocol. The current decay was expressed as the time necessary for the current to decrease to 50% of its peak value (Τ_0.5_). In a subset of experiments, we used 40 μM Zn^2+^ (as ZnCl_2_) to block Zn^2+^-sensitive extrasynaptic GABA_A_ receptors [18].

### 2.4. Statistical Analysis 

Data are reported as mean ± s.e.m. Unless otherwise specified, numbers (n) refer to the oocytes used in each experiment. Before data analysis, normal distribution was assessed with a Shapiro–Wilk test and, according to the result, parametric (Student’s *t*-test,) or non-parametric (Wilcoxon signed rank test, Mann–Whitney rank sum test) tests were used, performed with Sigmaplot 15 software. Differences between two datasets were considered significant when *p* < 0.05, two-tailed.

## 3. Results

### 3.1. GABA_A_ Current Rundown

Here, we performed different electrophysiological experiments using total membranes or synaptosomes from three TLE patients. Application of 500 μM GABA evoked currents with amplitudes very similar in both the samples (ranging from −18 to 390 nA), but with a large variability depending on the frog and/or the level of expression of the oocytes.

In one set of experiments, we analysed the receptor’s desensitization (i.e., rundown) using total membranes and synaptosomes from the same cortical samples of TLE patients (#1 and #2, Table 1).

**Figure 1:**
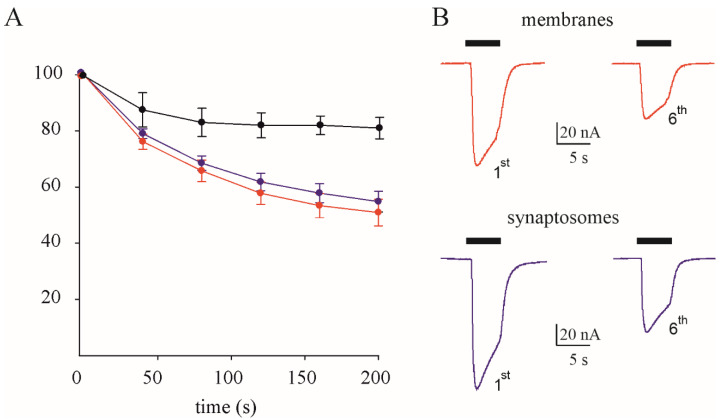
GABA current rundown in membranes versus synaptosomes. Properties of I_GABA_ rundown in membranes and synaptosomes. (**A**) I_GABA_ rundown as percentage of the first GABA application (●, membranes from one control individual; n = 8; ●, membranes from TLE patients; n = 12; ●, synaptosomes from the same TLE patients; n = 11; #1–2, Table 1). (**B**) Representative currents (first and sixth application) from the same experiments.

The GABA current rundown did not show significant changes between the total membranes (50.9 ± 4.7 %, n = 12, Figure 1) and synaptosomes (54.8 ± 3.7 %, n = 11, Figure 1), *p* = 0.56, nor did the GABA current decay (T_0.5_ = 9.6 ± 0.19 s in membranes versus T_0.5_ = 9.5 ± 0.31 s in synaptosomes, *p* = 0.81, with 8 degrees of freedom). Notably, there was a negligible current rundown in oocytes injected with control membranes from an individual without neurological disorders (81.25 ± 3.8%, n = 8, Figure 1), as previously reported [28]. This result reinforces our previous hypothesis that I_GABA_ rundown is a hallmark of TLE, mostly due to synaptic phasic inhibition [18].

### 3.2. GABA Reversal Potential in Oocytes Injected with Membranes and Synaptosomes

**Figure 2:**
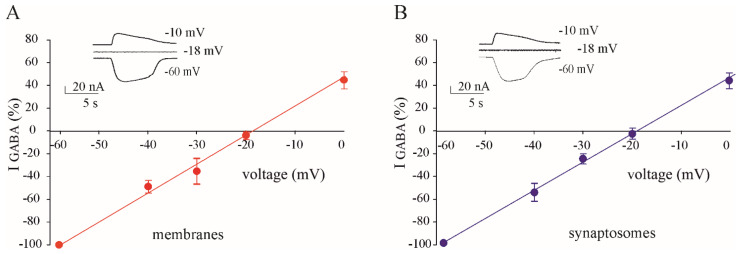
GABA reversal potential (E_GABA_) in membranes versus synaptosomes. E_GABA_ in oocytes injected with total membranes ((**A**) ●, n = 8) or with synaptosomes (**B**) ●, n = 8) from the hippocampal subicula of TLE patients (#1–2, Table 1). Each dot represents mean ± s.e.m. as a percentage of the current evoked at a holding potential = 60 mV. (Inset) Representative currents from the same experiments.

To better characterise any difference in GABAergic function in our samples, we carried out E_GABA_ recordings in oocytes injected with membrane and synaptosomes from the same TLE patients. Firstly, we used samples from the temporal lobe cortex (#1,2; Table 1) and found that the E_GABA_ did not change between total membranes and synaptosomes (E_GABA_ = −24.10 ± 0.8 mV, n = 7; E_GABA_ = −24.4 ± 0.60 mV, n = 7 for membranes and synaptosomes, respectively). Notably, these values are very similar to those previously published using tissues from other TLE patients and control tissues [20]. Secondly, we used samples of hippocampal subiculum (#3; Table 1) and we demonstrated a depolarized E_GABA_ that was not statistically different between total membranes and synaptosomes. (E_GABA_ = −18.20 ± 1.0 mV, n = 8; E_GABA_ = −18.90 ± 0.75 mV, n = 8 for membranes and synaptosomes, respectively, Figure 2.) Altogether, our results support the idea that the altered chloride homeostasis in human subicula from TLE patients is mainly localized at the synapse.

### 3.3. Lack of Inhibition of GABA_A_Rs in Synaptosomes after Zn^2+^ Application

**Figure 3:**
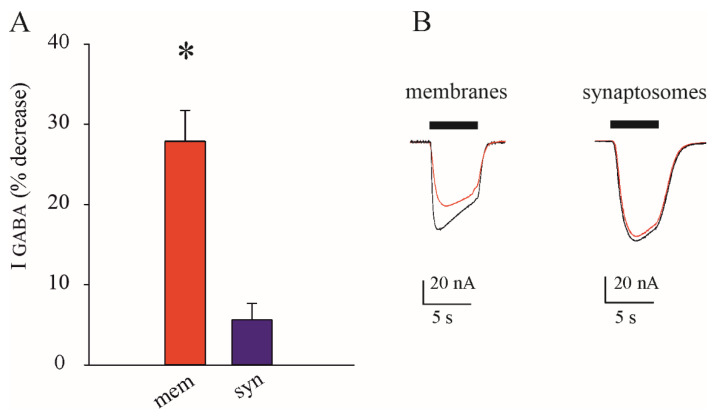
Zn^2+^ selectively inhibits extrasynaptic GABA_A_Rs. (**A**) Bars represent the GABA current (as %) decrease ± s.e.m after treatment with Zn^2+^ in oocytes injected with total membranes (red, n = 10) and synaptosomes (purple, n = 10) from the cortical samples of TLE patients (#1–3, Table 1); asterisk indicates *p* < 0.001, Student’s *t*-test. (**B**) representative currents evoked in oocytes injected with total membranes and synaptosomes before (black traces) and after their treatment with Zn^2+^ (red traces).

Both the alterations of GABA_A_-mediated transmission described above are due to the synaptic GABA_A_ receptors. To further validate our microtransplantation of synaptosomes approach we performed further experiments using Zn^2+^, which selectively blocks γ-less extrasynaptic GABA_A_Rs [18,39]. Interestingly, in the same cortical samples of TLE patients (#1–#3, Table 1), we found that 40 μM of Zn^2+^ was able to significantly decrease the I_GABA_ amplitude in oocytes injected with total membranes (27.9 ± 3.8%, n = 10, *p* < 0.001, with 9 degrees of freedom, Figure 3), whereas its effect on those injected with synaptosomes was negligible (5.6 ± 2.1%, n = 10, *p* = 0.35, with 9 degrees of freedom, Figure 3). This last result confirms that synaptosomes are mostly enriched with Zn^2+^-insensitive γ-containing synaptic receptors.

## 4. Discussion

In this study, we took advantage of the isolation of synaptosomes to investigate whether the GABAergic impairment previously described [20,28] was related to a “synaptopathy”. This method allows for an enrichment of cellular components found at the synapses isolated from different types of samples (animal or human tissues), thus providing a useful model to study the function of channels, transporters, or receptors localized to the synaptic area [26]. In recent years, we have studied GABAergic function, mostly in human epileptic tissues, using the “microtransplantation technique” in *Xenopus* oocytes [17,28]. Indeed, it was possible to record GABA-evoked currents using two-electrode voltage-clamp recordings in oocytes transplanted with tissues from epileptic patients, including those affected by rare diseases [19,40]. Among our relevant findings, we described for the first time a use-dependent desensitization (i.e., rundown) of GABA_A_ currents in patients affected by TLE that was absent in healthy brains [17,28]. This phenomenon was prevented by phosphatase inhibitors, and several agents enhancing phosphorylation processes [17,29]. However, we are aware that this procedure may have some limitations, such as the mixed compositions of the membranes used (i.e., glial and neuronal membranes) and the impossibility of distinguishing synaptic from extrasynaptic compartments. Here, total membranes isolated from the temporal cortex yielded I_GABA_ rundown values similar to those previously published by our group [18,41]. Notably, this value remained the same when we used synaptosomes from the same TLE patients, indicating that this phenomenon is mostly due to synaptic GABA_A_-Rs. Indeed, earlier reports [17,18] showed that, in human TLE, I_GABA_ rundown was generated by the repetitive activation of low-affinity phasic GABA_A_ receptors that are mostly localized to the synaptic cleft. Thus, we can hypothesize that the phosphorylation of GABA_A_ receptor subunits or their associated proteins targets mainly synaptic mechanisms [42,43]. Further studies will better elucidate this point. Another interesting point of discussion is the alteration of the chloride homeostasis which may occur in many epileptic conditions. Indeed, there are many relevant findings supporting the fact that GABA can behave as “less hyperpolarizing” and thus “less inhibitory”, exacerbating the severity of seizures [32,44]. Therefore, the alteration of the expression of the chloride transporters NKCC1 and KCC2 has been clearly demonstrated in human epileptic tissues [19,20,45], but the localization of these two transporters is not completely clear. In this study, we found an altered E_GABA_ both in total membranes and synaptosomes from epileptic subicula, indicating that chloride “dyshomeostasis” is confined mostly to the synaptic area. This finding may acquire a pathogenic relevance, since fast synaptic inhibition due to the release of a high GABA concentration [30] may be strongly affected by this chloride unbalance, especially during the firing of underlying recurrent seizures. To further elucidate this point, experiments using human slices from drug-resistant patients could be extremely useful. However, this approach may be limited by fresh human tissues’ availability.

Synaptosomes are known to be enriched with synaptic fractions [26,27]. To further reinforce this notion, adding some functional correlates, we used Zn^2+^ to selectively block extrasynaptic γ-less GABA_A_ receptors [8]. Here, by applying 40 µM of Zn^2+^ to oocytes injected with total membranes we found a decrease of about 28% of the GABA current’s amplitude compared to the negligible decrease in oocytes injected with synaptosomes from the same TLE patients. This last finding strengthens the validity of our approach to distinguishing synaptic GABA_A_-evoked currents.

In conclusion, using a combination of simple but powerful approaches, we highlight two main points: (1). the possibility of improving the microtransplantation technique by focusing on selected subtypes of cell membranes, and (2). the GABAergic deficits previously shown in human TLE tissues are mostly disturbing physiological equilibrium at the synapse.

## Figures and Tables

**Table 1:** Demographic and clinical characteristics of TLE patients.

Patient	Age (y)/Gender	Epilepsy Onset (y)	Seizure Types	Pathology	Surgical Zone	ASMs
#1	27/M	10	FIAS	HS	Right temporal	CBZ; TPM
#2	26/M	6	FIAS	HS	Left temporal	LEV; LMT; TPM
#3	28/F	14	FAS	HS	Left temporal	CBZ; LMT

Abbreviations: M, male; F, female; HS, hippocampal sclerosis; ASMs, Anti-Seizure Medications; CBZ, Carbamazepine; LEV, levetiracetam; LMT, lamotrigine; TPM, topiramate; FIAS, focal impaired awareness seizures; FAS, focal aware seizures.

## Data Availability

The datasets used and/or analysed during the current study are available from the corresponding author upon reasonable request.
